# Dental Staff Members’ Perception Regarding Objective Structured Clinical Examination (OSCE): An Experience from Saudi Arabia

**DOI:** 10.3390/dj13090423

**Published:** 2025-09-14

**Authors:** Ahmad A. Alnazzawi

**Affiliations:** Department of Substitutive Dental Sciences, College of Dentistry, Taibah University, Al-Madinah 42353, Saudi Arabia; anazawi@taibahu.edu.sa

**Keywords:** OSCE, dental education, faculty perception, assessment methods, validity, reliability, Saudi Arabia

## Abstract

**Background:** The Objective Structured Clinical Examination (OSCE) is a widely used assessment tool in education in the health profession. Understanding faculty perceptions of the OSCE regarding fairness, validity, reliability, and effectiveness is essential to enhancing its implementation in dental education in Saudi Arabia. **Objective:** To assess dental faculty members’ perceptions of the OSCE as an assessment method in Saudi Arabia, focusing on its fairness, reliability, validity, and effectiveness compared to other assessment formats. **Methods:** A cross-sectional analytical survey-based study was conducted among dental faculty members across 11 dental colleges in Saudi Arabia. A modified validated questionnaire was distributed electronically through the college deans to faculty members. Responses were analyzed using SPSS v25, applying descriptive and inferential statistics (chi-square test, significance set at *p* ≤ 0.05). **Results:** A total of 181 faculty responses from 11 Saudi dental colleges indicated broad support for the OSCE as a fair (78.3%), well-structured (71.1%), and comprehensive assessment of clinical skills (70%). While nearly half found it less stressful than other formats, 30% still perceived it as intimidating. Faculty agreed that OSCE tasks reflected taught content (65.9%), instructions were clear (75.7%), and scores were largely standardized and unbiased (63.9–72.9%). Compared with other assessment methods, essays/ short-answer questions (SAQs) were viewed as most effective for learning, but the OSCE was most favored for continued use in clinical training (52.8%). **Conclusions:** Faculty members in Saudi dental schools generally view the OSCE as a fair, valid, and effective assessment method. Despite concerns about student preparedness and perceived difficulty, the OSCE is endorsed as a key tool for evaluating clinical competencies. Addressing these concerns may further enhance its educational value and implementation.

## 1. Introduction

Assessment methods in dental education play a crucial role in evaluating students’ clinical competencies and ensuring their readiness for professional practice [1]. Among these methods, the Objective Structured Clinical Examination (OSCE) has gained widespread recognition as a standardized and practical approach to assessing clinical skills, decision-making abilities, and overall competence in healthcare education [2]. The OSCE evaluates various competencies through structured stations, each focusing on specific clinical tasks, thereby ensuring a comprehensive and fair assessment [3,4,5].

Traditional assessment methods, such as multiple-choice questions (MCQs), short-answer questions (SAQs), and clerkship ratings, are commonly used in dental education. However, these formats often fail to capture the complex and multifaceted nature of clinical performance and practical skills [6]. In contrast, the OSCE offers a structured environment that enhances objectivity and reduces examiner bias [7]. Despite its advantages, concerns have been raised about its perceived difficulty, fairness, and impact on stress levels among both faculty and students [8]. Understanding faculty perceptions of the OSCE is essential for improving its implementation and ensuring that it remains a reliable and valid assessment tool [2,3].

Faculty perceptions play a crucial role in shaping the design, implementation, and long-term success of OSCEs. Research from multiple countries consistently highlights positive faculty attitudes toward OSCEs. For example, Wali et al. (2021) [9] found that dental faculty members overwhelmingly supported the OSCE, recognizing it as a reliable and indispensable tool within competency-based education, particularly for evaluating cognitive and diagnostic skills. Similarly, international studies have underscored the educational value and perceived fairness of OSCEs. Alsaid and Al-Sheikh (2017) [10], for instance, reported that 80% of faculty considered the OSCE a superior assessment method compared to traditional examinations. Fouad et al. (2019) [11] emphasized that faculty members identified training and standardization as critical factors for enhancing the validity of OSCEs and minimizing examiner bias. In Jordan, Jallad et al. (2023) [12] observed that over 82% of nursing faculty held positive perceptions of the OSCE as a vital and objective method for assessing students’ clinical competence.

While research from multiple countries consistently highlights positive faculty attitudes toward OSCEs, to the best of our knowledge, no large-scale studies have examined the perception of Saudi teaching staff. This apparent gap in the literature suggests the need for a more comprehensive investigation in the Saudi context. Understanding local faculty perspectives, challenges, and contextual factors is essential for optimizing the use and effectiveness of the OSCE in Saudi dental education.

Several faculty members expressed concerns regarding the standards or quality of OSCEs implemented as an assessment tool, despite their understanding of the evidence supporting its effectiveness. This study aims to assess faculty perceptions of the OSCE in dental education in Saudi Arabia, with a focus on its fairness, reliability, validity, and effectiveness compared to other assessment methods. It is hypothesized that dental faculty members perceive the OSCE as a fair, reliable, and valid method of assessment, but may also express concerns about its level of difficulty and students’ preparedness for the examination.

## 2. Materials and Methods

### 2.1. Study Design

This study employed a cross-sectional analytical observational design utilizing a survey-based approach to collect data that are relevant to the research objectives. This methodological framework provides a foundation for enhancing existing programs and interventions [13].

### 2.2. Study Setting and Sample

The study was conducted in Saudi Arabia and targeted dental staff members from various colleges of dentistry nationwide. A non-probability convenience sampling technique was used to recruit participants. Electronic questionnaires were disseminated to the deans of 20 dental colleges across the kingdom, who were then requested to distribute them among their respective staff members.

### 2.3. Data Collection Instrument

The questionnaire was administered in Arabic and included demographic information alongside standardized assessment tools. The primary instrument used was a modified self-administered version of a questionnaire developed by Pierre, Wierenga, Barton, Branday, and Christie (2004) [14]. This questionnaire is a validated and reliable tool, with translation and validation conducted by Al Zeftawy et al. (2016) [15].

The key outcome measures assessed in the questionnaire included staff members’ perceptions of the attributes of the Objective Structured Clinical Examination (OSCE). These attributes encompassed the quality of instructions and organization, the quality of performance, and the perceived usefulness of the OSCE compared to other assessment methods. Responses were recorded using a four-point Likert scale to measure levels of agreement on most dimensions. Additionally, using a three-point scale, the OSCE was evaluated in comparison to other assessment formats based on difficulty, fairness, learning outcomes, and preferred frequency of use.

### 2.4. Ethical Considerations

Ethical approval for this study was obtained from the TUCD Research Ethics Committee (TUCD-REC) under approval number TUCDREC/20170404/AlNazzawi. TUCD-REC granted a waiver of documentation of informed consent. To ensure ethical compliance, a cover letter detailing the study’s objectives and emphasizing voluntary participation was included at the beginning of the questionnaire. Participants were assured that their responses would remain confidential and anonymous. Those who consented to participate completed an electronic self-administered questionnaire to assess their perceptions of the OSCE as an assessment strategy.

### 2.5. Data Analysis

Statistical analyses were performed using SPSS software, version 25. Data were coded, analyzed, and presented in tabular format. Both descriptive and inferential statistical methods were employed, including the chi-square test. A significance level of *p* ≤ 0.05 was considered to control for Type I error.

## 3. Results

A total of 181 responses were received from 11 dental colleges across Saudi Arabia, yielding an overall institutional participation rate of 55% (11 out of 20 colleges). The participating institutions included Taibah University, Taif University, King Abdulaziz University, King Saud University, King Khalid University, Najran University, Al Jouf University, Farabi Colleges, Riyadh, Ibn Sina National College for Health Sciences, Jazan University, and Imam Abdulrahman Bin Faisal University (formerly Dammam University). Although the individual response rate per college could not be determined due to the distribution method, the broad representation across different institutions enhances the generalizability of the findings, while also highlighting the need for cautious interpretation given the non-probability sampling approach. These institutions comprised nine governmental and two private dental colleges. Among the respondents, 136 were male (75%), while 45 were female (24.9%). All dental specialties were represented, with the highest participation from Restorative Dental Sciences (23.8%), followed by Basic and Clinical Oral Sciences (19.3%), and Preventive Dental Sciences (16.6%). The remaining departments contributed between 12% and 13% of the total sample.

### 3.1. OSCE Evaluation

As illustrated in Table 1, a significant proportion of dental faculty members (78.3%) expressed agreement regarding the fairness of the OSCE. Two-thirds (66.7%) also deemed it to encompass a comprehensive range of knowledge. In terms of time allocation, 40% of respondents felt that additional time was necessary at the assessment stations, while 31.1% maintained a neutral stance, and 23.9% disagreed, indicating a diverse array of perspectives. A considerable majority (69.4%) reported that the examination was administered effectively, and 71.1% concurred that it was well-structured and logically sequenced.

Concerning stress levels, only 26.1% of participants identified the examination as highly stressful, whereas 47.2% perceived the OSCE as less stressful in comparison to other assessment formats. Nevertheless, 30% of respondents regarded the OSCE as intimidating. Notably, 67.8% agreed that the OSCE provided students with opportunities to compensate in certain areas, and 61.1% recognized that it facilitated the identification of students’ weaknesses.

Perceptions regarding student preparedness were varied: 61.7% of faculty members believed that students were cognizant of the level of knowledge required for the OSCE, while a significant minority (23.3%) remained neutral. Importantly, 70% of respondents concurred that the OSCE effectively encompassed a wide array of clinical skills.

### 3.2. Evaluation of OSCE Performance

Table 2 presents the faculty members’ assessment of the quality of OSCE performance. More than half of the respondents (60.2%) strongly agreed that students were fully aware of the nature of the examination. Additionally, 65.9% believed that the procedures assessed in the OSCE reflected the content taught during the course. The allotted time per station was considered adequate by 63.3% of the participants. Furthermore, 57.2% of faculty members agreed that the setting, sequence, and context of each OSCE station were authentic.

A high proportion of respondents also agreed that the instructions provided were clear, the tasks assigned were fair, the sequencing of stations was logical, and the OSCE offered valuable learning opportunities. The percentage of agreement for these aspects ranged from 67.2% to 75.5%.

### 3.3. Perception of Validity and Reliability

Table 3 illustrates faculty members’ perceptions of the validity and reliability of the OSCE. A substantial proportion of respondents agreed to a great extent that OSCE scores were standardized (63.9%) and that the exam was practical and useful (74.7%). Furthermore, 72.9% believed that factors such as personality, ethnicity, and gender did not influence OSCE scores.

The lowest proportion of agreement in this category was related to whether OSCE scores provided a true measure of essential clinical skills in dentistry. However; more than half (58%) still agreed with this statement.

### 3.4. Comparison of Assessment Formats

Table 4 presents faculty members’ evaluations of different assessment formats, including MCQs, SAQs/essays, OSCEs, and clerkship ratings. The majority of faculty members (67.2%) identified MCQs as the easiest format, while only 13.9% selected OSCEs. However, the observed differences between male and female faculty in their choices were not statistically significant (χ^2^ = 2.4, *p* = 0.492), indicating that gender did not influence perceptions regarding the easiest assessment format.

When asked which format was the fairest, 36.7% of respondents selected MCQs, followed by SAQs/essays (31.7%) and OSCEs (30%). Clerkship ratings were least frequently identified as fair (1.7%). Again, no significant gender-based differences were found (χ^2^ = 3.8, *p* = 0.280).

Regarding the format that is most effective in promoting student learning, 43.3% of faculty favored SAQs/essays, while 33.9% selected OSCEs and 18.9% selected MCQs. Although female faculty appeared to be slightly more likely to select essays/SAQs (50% vs. 41.2%), this difference was not statistically significant (χ^2^ = 2.41, *p* = 0.492).

Finally, 52.8% of faculty members expressed support for the continued use of OSCEs in clinical years, compared to 30% for essays/SAQs and 12.2% for MCQs. Despite these preferences, no significant gender-based differences were observed (χ^2^ = 3.786, *p* = 0.285).

Overall, while descriptive results highlight clear preferences for MCQs as the easiest format and OSCEs as the most valuable in clinical years, none of the differences between male and female faculty members reached statistical significance. This suggests that faculty perceptions of assessment formats were consistent across gender groups.

## 4. Discussion

The findings of this study offer valuable insights into dental faculty perceptions of the OSCE as an assessment method within Saudi Arabia, aligning with and expanding upon results from both the local and international literature. Overall, the faculty in this study viewed the OSCE as a fair, structured, and relevant assessment tool—consistent with previous findings by Wali et al. (2021) [9], who reported that Saudi dental faculty widely endorsed the OSCE’s role in competency-based education and the evaluation of clinical skills.

It is noteworthy to compare the current study’s findings with students’ perceptions of the OSCE in Saudi Arabia. Al Nazzawi (2018) [16] reported that only 47.1% of dental students considered the OSCE to be fair, and a majority perceived it as stressful and intimidating (62.2%). Furthermore, students questioned its validity and reliability, with only 29.4% agreeing that it provides a true measure of essential clinical skills. In contrast, the present study demonstrated that faculty members overwhelmingly endorsed the OSCE as a fair (80.1%), valid (72.9%), and reliable (63.9%) assessment method. These differences highlight a perceptual gap between faculty and students, particularly regarding fairness and stress, which may be attributed to differences in experience, expectations, and preparedness. Such divergence emphasizes the importance of bridging faculty and student perspectives in order to optimize the educational value of the OSCE and address concerns related to stress management, preparation, and transparency in assessment criteria.

### 4.1. Fairness and Comprehensiveness of the OSCE

A large majority of faculty members in the present study (78.3%) regarded the OSCE as a fair examination, closely aligning with the findings of Alsaid and Al-Sheikh (2017) [10], where 80% of medical faculty considered the OSCE to be a fairer assessment method than traditional long- and short-case exams. This perceived fairness is largely attributed to the OSCE’s structured stations, which help minimize examiner bias—an advantage [17] also highlighted by Khan et al. (2022) [5], who reported that 76% of faculty in Pakistan viewed the OSCE as more objective than conventional clinical exams. Furthermore, 66.7% of faculty in the current study agreed that the OSCE covered a broad spectrum of knowledge, echoing the findings of Fouad et al. (2019) [11], in which 83% of participants valued the OSCE as a comprehensive and practical learning experience. However, concerns about student preparedness—reflected in the finding that 62.4% of faculty believed students were not fully aware of the required knowledge—align with Fouad et al.’s emphasis on the need for targeted OSCE training and preparation strategies [11]. These findings suggest a need for more effective pre-exam orientation, as supported by Lee et al. (2018) [18], who demonstrated that student-led mock OSCEs significantly improved confidence and performance. Collectively, this highlights a recurring challenge: bridging the gap between assessment expectations and student readiness [19,20,21].

### 4.2. Faculty Perceptions of OSCE Performance and Administration

The majority of faculty members in this study acknowledged the smooth administration of the OSCE (69.4%) and its logical sequencing (93.4%). Additionally, 63.3% approved of the time allocated per station, and 57.2% affirmed the authenticity of the clinical scenarios. These findings are consistent with those of Wali et al. (2022) [9], who emphasized the importance of structured implementation and the use of standardized patients. Similarly, Jallad et al. (2024) [12] reported strong agreement among nursing faculty (82.2%) that OSCE stations should reflect real clinical situations, further underscoring the importance of realism in assessment. However, the perception of the OSCE as intimidating for students—reported by 30% of faculty in this study—echoes concerns raised by Fouad et al. (2019) [11] regarding OSCE-related stress. This finding is also consistent with that of Hosseini F. A. et al. (2025), who attributed OSCE-related stress to its high-stakes nature and time constraints [19]. To address this issue, Barman proposed integrating formative OSCEs with low-stakes feedback, which was shown to reduce anxiety and improve performance in a Malaysian medical school cohort [22]. Collectively, these findings highlight the need for enhanced student support strategies, such as mock exams, orientation sessions, and formative feedback [19,21,23,24,25].

### 4.3. Validity and Reliability of OSCE Scores

This study positively endorsed the reliability and objectivity of OSCE scores, with 63.9% of respondents agreeing on score standardization and 72.9% rejecting the influence of demographic biases. These findings are consistent with those of Wali et al. (2021) [9] and Fouad et al. (2019) [11], both of whom emphasized the OSCE’s role in mitigating examiner bias through structured criteria and faculty calibration. However, the fact that only 58% of respondents believed OSCE scores accurately reflect essential clinical skills indicates a shared concern across studies regarding the OSCE’s limitations in comprehensively capturing clinical competence. This suggests a need for further refinement of scoring rubrics and station design [26,27]. Similar challenges were highlighted during the adaptation of OSCEs under COVID-19 conditions, where the importance of sound educational principles such as blueprinting, clear rubrics, multiple examiners, and structured feedback was emphasized to enhance validity and fairness [28]. These findings suggest that further refinement of scoring rubrics, the incorporation of detailed checklists, and the use of collaborative examiner calibration are necessary to strengthen objectivity. Moreover, integrating structured feedback and formative elements into OSCEs may not only improve scoring reliability but also enhance students’ preparedness and confidence, thereby addressing concerns about the comprehensiveness of OSCE-based assessment.

### 4.4. Comparison with Other Assessment Formats

Compared to other assessment formats, the OSCE was perceived as more demanding than MCQs, with 67.2% of faculty considering MCQs the easiest. Nevertheless, the OSCE was viewed as superior in evaluating clinical skills. This perspective aligns with Alsaid and Al-Sheikh (2017) [10], who noted faculty preference for OSCEs over traditional examinations due to their practical orientation. However, the recognition that essays and SAQs were better facilitate learning supports the notion—highlighted in Fouad et al. (2019) [11]—that a diverse array of assessment methods should be integrated to ensure a balanced evaluation of both theoretical knowledge and practical skills [29,30,31].

Furthermore, the study found that 52.8% of faculty supported the use of OSCEs in clinical years, confirming its perceived value in dental education. Additionally, the absence of significant gender-based differences in perception reflects findings from Jallad et al. (2023) [12], where variations were more influenced by experience and educational background than by gender. These findings affirm the OSCE’s growing role in health profession education and signal the need for ongoing improvements tailored to the Saudi context.

The absence of statistically significant gender-based differences across all assessment formats indicates that faculty perceptions were broadly consistent regardless of gender. This suggests that preferences for certain assessment methods, such as favoring MCQs for ease or OSCEs for clinical application, are likely influenced more by educational philosophy and teaching experience than by demographic factors.

A study was conducted by Wali et al. (2022) [9], who evaluated faculty perceptions of the OSCE within a smaller sample of 101 respondents from seven institutions across Saudi Arabia. While their study provided valuable insights into faculty knowledge, attitudes, and practices regarding OSCE implementation, our investigation expands the scope considerably by including a larger and more diverse sample of 181 faculty members from 11 dental colleges nationwide, thereby offering a broader perspective. In addition, whereas Wali et al.’s study focused primarily on the logistical and curricular aspects of OSCE use, our study examined perceptions related to fairness, reliability, validity, and stress in comparison to other assessment methods, allowing for more direct alignment with parallel student-based research in Saudi Arabia. This dual contextualization provides a novel contribution by highlighting the perceptual gap between educators and learners.

### 4.5. Limitations

One limitation of this study is that the questionnaire used was originally designed to assess student perceptions of the OSCE and was later adapted for use with faculty members. As such, certain items—such as stress during the exam or students’ awareness of exam content—may not be directly measurable from the faculty perspective and should be interpreted with caution.

### 4.6. Implications and Recommendations

The findings of this study underscore the need for the continuous refinement of the OSCE to enhance its effectiveness as an assessment tool. Addressing student preparedness through pre-exam workshops, providing detailed feedback, and incorporating additional support mechanisms can improve students’ experiences and performance. Moreover, ensuring that OSCE stations comprehensively cover essential clinical skills can further strengthen its validity and reliability.

Future research should explore students’ perspectives on the OSCE to provide a more holistic understanding of its impact on learning outcomes. Additionally, investigating potential modifications to assessment criteria and station design can help optimize the OSCE as a gold standard in clinical evaluation. Moreover, future studies should consider developing or validating instruments that are specifically tailored for teaching staff to capture their unique perspectives more objectively. Additionally, a mixed-methods approach—combining faculty surveys with student feedback and qualitative interviews—would allow for a more comprehensive evaluation of OSCE implementation and help bridge the perceptual gap between educators and learners.

## 5. Conclusions

In conclusion, this study provides compelling evidence that the OSCE is a fair, valid, and well-structured assessment method in dental education. Faculty members generally perceive it as an effective tool for evaluating clinical competencies, despite some concerns regarding student preparedness and the perceived difficulty of the examination. By addressing these concerns through targeted interventions, the OSCE can be further improved to ensure that it remains a robust and equitable dental education assessment method.

## Figures and Tables

**Table 1:** Dentistry Staff members’ Evaluation of OSCE attributes (N= 181):.

	Question	Agree		Disagree		Neutral		No Comment	
		N	%	N	%	N	%	N	%
1	Exam was fair	141	78.3	6	3.3	29	16.1	5	2.8
2	Wide knowledge area covered	120	66.7	17	9.4	43	23.9	1	0.6
3	Needed more time at stations	72	40.0	43	23.9	56	31.1	10	5.6
4	Exams well administered	125	69.4	13	7.2	40	22.2	3	1.7
5	Exams very stressful	47	26.1	72	40.0	56	31.1	6	3.3
6	Exams well-structured and sequenced	128	71.1	7	3.9	40	22.2	6	3.3
7	Exam minimized chance of failing	66	36.7	36	20.0	76	42.2	3	1.7
8	OSCE is less stressful than other exams	85	47.2	48	26.7	47	26.1	1	0.6
9	Allowed student to compensate in some areas	122	67.8	18	10.0	38	21.1	3	1.7
10	Highlighted areas of weakness	110	61.1	21	11.7	46	25.6	4	2.2
11	Exam intimidating	54	30.0	39	21.7	76	42.2	12	6.7
12	Student not fully aware of level of information needed	111	61.7	24	13.3	42	23.3	4	2.2
13	Wide range of clinical skills covered	126	70.0	26	14.4	15	8.3	14	7.8

**Table 2:** Dentistry Staff members’ Evaluation of the quality of OSCE performance (N= 181).

	Question	To Great Extent		Neutral		Not at All	
		N	%	N	%	N	%
1	Students were fully aware of nature of exam	109	60.2	59	32.6	13	7.2
2	Tasks reflected those taught	118	65.9	58	32.4	3	1.7
3	Usually time at each station was adequate	114	63.3	56	31.1	10	5.6
4	Setting and context at each station felt authentic	103	57.2	72	40	5	2.8
5	Instructions were clear and unambiguous	137	75.7	41	22.7	3	1.7
6	Tasks asked to perform were fair	122	68.2	51	28.5	6	3.4
7	Sequence of stations logical and appropriate	121	67.2	55	30.6	4	2.2
8	Exam provided opportunities to learn	128	70.7	44	24.3	9	5

**Table 3:** Dentistry staff members’ perception of validity and reliability of OSCE scoring and objectivity.

	Question	To Great Extent		Neutral		Not at All	
		N	%	N	%	N	%
1	OSCE exam scores provide true measure of essential clinical skills in dentistry	105	58	61	33.7	15	8.3
2	OSCE scores are standardized	115	63.9	57	31.7	8	4.4
3	OSCE practical and useful experience	133	74.7	43	24.2	2	1.1
4	Personality, ethnicity and gender will not affect OSCE scores	132	72.9	37	20.4	12	6.6

**Table 4:** Dentistry staff members’ rating for assessment formats.

	Question	Total		Male		Female		X^2^ *p*-Value
		N = 180	%	N = 136	%	N = 44	%	
1	Which of the following formats is easiest?							
	MCQ	121	67.2	92	67.6	29	65.9	X^2^ = 2.4 *p* = 0.492 *p* > 0.05 (NS)
	Essay/SAC	29	16.1	22	16.2	7	15.9	
	OSCE	25	13.9	17	12.5	8	18.2	
	Clerkship rating	5	2.8	5	3.7	0	0.0	
2	Which of the following formats is fairest?							
	MCQ	66	36.7	52	38.2	14	31.8	X^2^ = 3.8 *p* = 0.28 *p* > 0.05 (NS)
	Essay/SAC	57	31.7	41	30.1	16	36.4	
	OSCE	54	30	42	30.9	12	27.3	
	Clerkship rating	3	1.7	1	0.7	2	4.5	
3	From which of the following formats do you expect students learn most?							
	MCQ	34	18.9	29	21.3	5	11.4	X^2^ = 2.41 *p* = 0.492 *p* > 0.05 (NS)
	Essay/SAC	78	43.3	56	41.2	22	50	
	OSCE	61	33.9	46	33.8	15	34.4	
	Clerkship rating	7	3.9	5	3.7	2	4.5	
4	Which of the following formats should be used more often in the clinical years of the program?							
	MCQ	22	12.2	17	12.5	5	11.4	X^2^ = 3.786 *p* = 0.285 *p* > 0.05 (NS)
	Essay/SAC	54	30	38	27.9	16	36.4	
	OSCE	95	52.8	76	55.9	19	43.2	
	Clerkship rating	9	5	5	3.7	4	9.1	

## Data Availability

All data ar included in this manuscript.
